# Primary non-Hodgkin’s lymphoma of the mandible

**DOI:** 10.4103/0973-029X.72506

**Published:** 2010

**Authors:** J Dinakar, Lakshmi Priya, Samyukta Reddy

**Affiliations:** *Department of Oral Pathology, Sri Ramakrishna Dental College and Hospital, Coimbatore, India*

**Keywords:** Immunohistochemistry, mandible, primary non-Hodgkin’s lymphoma

## Abstract

Primary non-Hodgkins’s lymphoma is a very uncommon lesion, accounting for 0.6% in jaws. As the lesions frequently resemble other disease such as chronic osteomyelitis, odontogenic or any secondary neoplasms, further evaluation and histopathologic examination allow early identification for appropriate treatment. The purpose of this case report is to describe a rare case of non-Hodgkin’s lymphoma of the mandible, explore the diagnosis and workup based on immunohistochemistry.

## INTRODUCTION

Malignant lymphoma is a neoplastic process of the lymphopoietic portion of the reticulo-endothelial system. Most of them originate from B-lymphocytes. Lymphoma is the second common malignancy of head and neck.[1] Primary non-Hodgkin’s lymphoma (NHL) of the bone is rare, accounting for <5%, and that of the mandible is 0.6%.[2] In 1963, the term primary lymphoma of bone was introduced by Ivins and Dahlin. The etiology is unknown even though virus and immunosuppression are implicated. The most common manifestation is pain and swelling in the jaw bone, but it is often clinically diagnosed as a dental infection. Hence, the diagnosis of lymphoma in jaw bone is often delayed. Biopsy is considered when there is a non-healing extraction wound and if any extra-oral wound is not resolving for a prolonged period. The histopathological diagnosis has to be confirmed by immunohistochemistry.

The WHO diagnostic criteria for primary lymphoma of bone are a primary focus in a single bone and histologic confirmation and, at the time of diagnosis, no evidence of distant soft tissue or lympnode involvement. There are no pathognomonic radiographic findings. Paresthesia along the inferior alveolar nerve distribution is a common finding, with reports ranging upto 20%. We present a case with a dental complaint which was later diagnosed as primary non-Hodgkin’s lymphoma of the mandible.

## CASE REPORT

A 54-year-old male presented with a past history of non-resolving extra-oral swelling on the right of the face. He underwent surgical removal of impacted right mandibular third molar 2 months back by an outside practitioner. He was under antibiotics and anti-inflammatory drug coverage for a few days. Following extraction, the swelling did not resolve and, subsequently, they extracted 47 and 46 presuming some dental infection.

**Figure 1:**
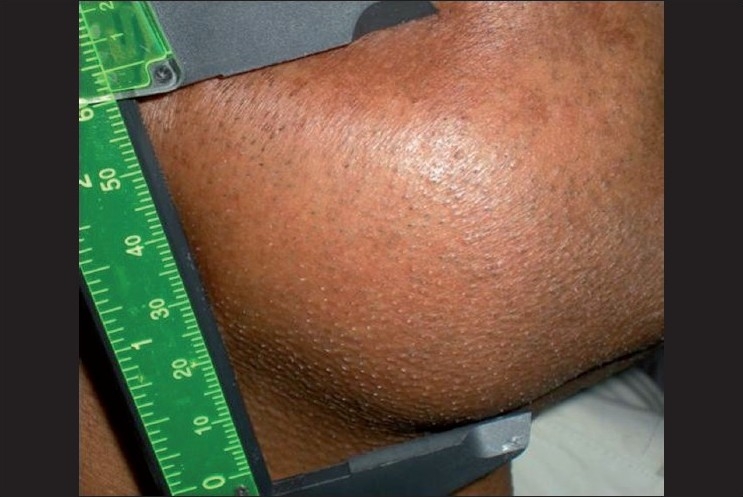
Diffus swelling on the right side of the face near the angle of the mandible

**Figure 2:**
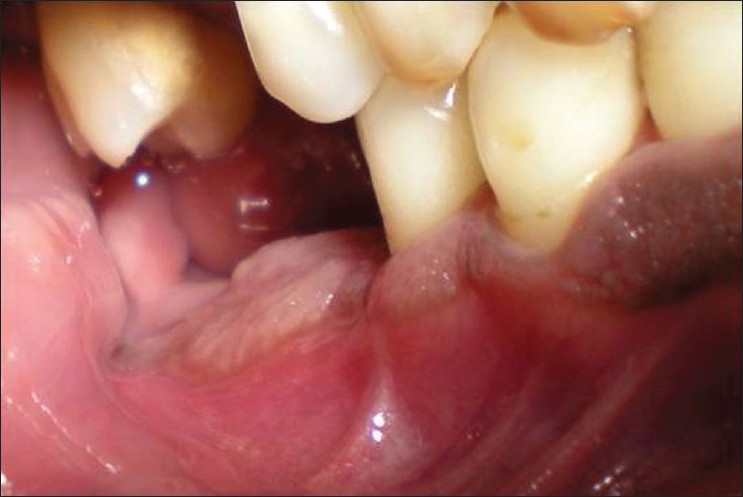
Intra-orally fleshy mass in the region of 46, 47 and 48 was seen

On extra-oral examination, a diffuse swelling was noted on the right side of the face near the angle of the mandible[Figure 1]. Intra-oral examination revealed a fleshy mass in the region of 46, 47 and 48. On palpation, the mass was tender[Figure 2]. Medical history was not contributory. Radiographically, minimal radiolucency in the 47 tooth region was seen. Based on the history and examination, a provisional diagnosis of “chronic osteomyelitis” was made. An intra-oral biopsy from the retromolar region was taken.

**Figure 3:**
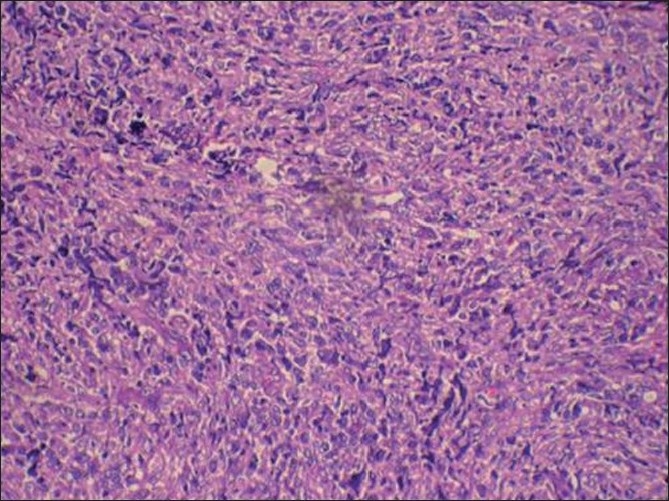
Hematoxylin and eosin staining (10×) showing sheets of atypical lymphoid cells

**Figure 4:**
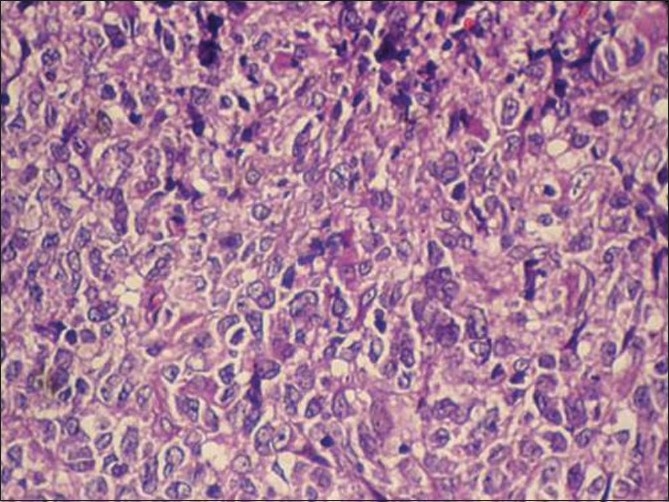
Hematoxylin and eosin (40×) section showing sheets of atypical lymphoid cells

**Figure 5:**
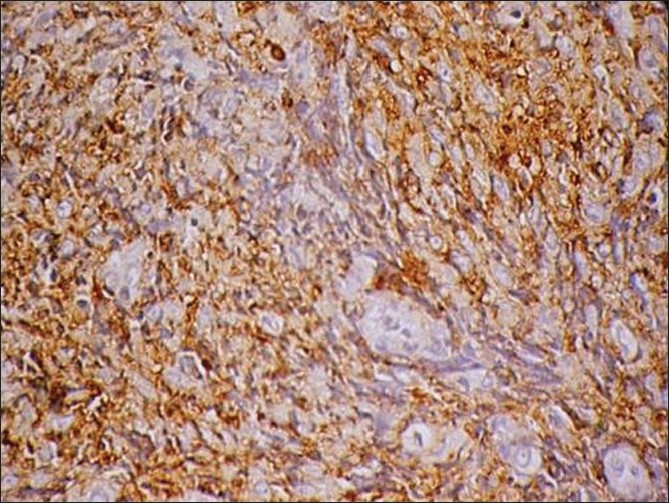
Immunohistochemistry profile showing positivity for LCA (CD 45)

**Figure 6:**
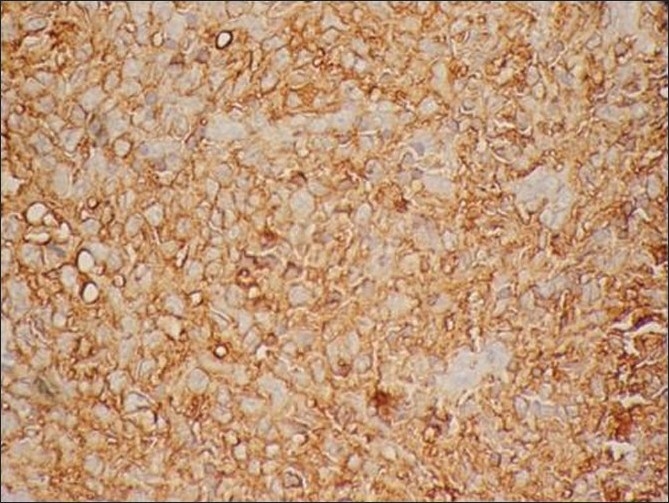
Immunohistochemistry profile showing positivity for CD 20

Histopathology showed sheets of atypical lymphoid cells. The individual cells were polyhedral with scant eosinophilic cytoplasm. The nuclei showed irregular membrane, open chromatin and prominent nucleoli. Foci of atypical mitotic figures were also seen. The lymphoid cells were seen infiltrating the bone. Areas of sclerosis, necrosis and hemorrhage were seen amidst these cells. Bony trabeculae, fibro-collagenous tissue and neural elements were seen [Figures 3 and 4]. The immunohistochemistry profile showed positivity for leucocyte common antigen (LCA) (CD 45)[Figure 5] and CD20[Figure 6]. Based on histopathology and immunohistochemistry, a diagnosis of non-Hodgkin’s lymphoma was made.

The patient’s general health was examined by a physician to find out involvement of any other site. Investigations like hemogram, radiograph and computed tomography (CT) scan were performed and no other site except the mandible showed involvement. Based on all the findings, a diagnosis of primary lymphoma of mandible was made. He was then referred to an oncologist for treatment.

## DISCUSSION

Primary non-Hodgkin’s lymphoma in extranodal sites accounts for 24–45% of the cases.[3,4] The common extranodal site is the gastrointestinal tract. The mandible accounts for only 0.6% of the isolated malignant non-Hodgkin’s lymphoma.[2] Whenever non-Hodgkin’s occurs in jaw bones, it is initially mistaken for a dental infection.[5,6] Thus, diagnosis of NHL is delayed. Many times, it is treated initially as dent-alveolar abscess or osteomyelitis.[7] A biopsy is performed only when there is no proper response to antibiotic treatment. The criteria for diagnosis of primary lymphoma of bone is suggested by Coley in 1950 with minor modifications as follows: lymphoma presenting in an osseous site with no evidence of disease elsewhere for at least 6 months after diagnosis.[5]

Primary bone lymphoma occurs in patients from 1 to 86 years (median range, 36–56 years) of age, with peak prevalence among patients in the 6^th^and 7^th^ decades of life. Our patient was in the 6^th^decade of life.

Most of the cases initially present as an odontogenic infection. Parrington *et al*. have discussed a case of primary lymphoma of the mandible presenting after tooth extraction.[8] Leva Dja vonmardi *et al*. have reviewed 16 cases of malignant non-Hodgkin’s lymphoma of the jaws and found that diagnosis that was usually difficult and was often misleading and delayed before the first bone biopsy.[9] Our case also demonstrated diagnostic difficulty by the dentist. Even the radiograph was not contributory to suspect a malignancy. There was not much of lytic destruction. Gusenbauer *et al*., in a case report of primary lymphoma of the mandible, emphasized that malignant lymphoma must be considered in the differential diagnosis of unexplained dental pain and swelling.[10] Bertolotto *et al*. have reported a case of primary lymphoma of the mandible with diffuse widening of the mandibular canal.[11] There are no pathognomonic radiographic findings. Features are usually that of non-specific osteolysis.

Usually, biopsy is carried out in the non-healing extraction site after repeated treatment for non-responding odontogenic infection. [12] Histopathology usually shows sheets of chronic inflammatory cells, especially lymphocytes, mimicking an inflammatory reaction. Careful examination by an expert histopathologist is necessary to find out the pleomorphic and atypical lymphoid cells. In our case, the histopathology showed sheets of lymphoid cells infiltrating the bone, with atypical mitotic figures, areas of sclerosis, necrosis and hemorrhage. Lymphoma was suspected and immunohistochemistry was performed for the following markers: LCA (CD45), CD15, CD20, CD30, PAN-CK, EMA, ALK1 and CD3. The result was positive for LCA (CD45)[Figure 5] and CD20 [Figure 6]. Therefore, based on the immunohistochemistry profile, a diagnosis of non-Hodgkin’s lymphoma was made.

When tissue diagnosis is confirmed as NHL of the mandible, determination must be made regarding orientation and spread of tumor. Overall assessment is essential to rule out nodal and visceral involvement.

A CT scan and Positron emission tomography (PET) scan can be carried out to look out for extranodal involvement. [13] Laboratory studies are not specific, although elevated lactate dehydrogenase is observed as a poor diagnostic factor.

In our case, we ruled out any other site involvement with a general physician. Investigations like hemogram, radiograph and CT scan were performed.

Accurate staging is essential to start the treatment and management. Staging takes into account the involved site and degree of dissemination. Primary NHL of bone as a single focus in an extranodal site is categorized as stage 1. Bachaud *et al*. have found that stage 1 NHL has a 5-year survival rate of 70%, and the median survival time for stage 1 is 10 years. The 5-year survival rate for stage 1 NHL of the maxillo-mandibular region is reported to be approximately 50%.[14]

The treatment of NHL is combination of surgery and chemotherapy-radiotherapy. In our case, after surgery, the patient was referred to an oncologist for chemotherapy and radiotherapy. The patient is responding well to the treatment. Beal *et al*., in a long-term follow-up of 82 patients with primary bone lymphoma, have found that patient’s prognosis is excellent, with a 5-year survival rate of 95%.[6]

Lymphoma of bone should be considered in the differential diagnosis of long-standing, non-healing extraction wound.
